# Distribution and efficacy of ingested dsRNA targeting tissue-specific genes in the Argentine ant, *Linepithema humile*

**DOI:** 10.1371/journal.pone.0323988

**Published:** 2026-07-28

**Authors:** Mathew A. Dittmann, Grzegorz Buczkowski, Brock A. Harpur

**Affiliations:** Department of Entomology, Purdue University, West Lafayette, Indiana, United States of America; University of Naples Federico II: Universita degli Studi di Napoli Federico II, ITALY

## Abstract

With the increasing availability of genomic and transcriptomic information within Formicidae, the investigation of gene function has become possible within ants. However, eusocial life history renders the generation of mutagenic strains of ants difficult outside of specific ant lineages that can induce reproductive behavior in workers. RNA interference (RNAi) remains a practical option to investigate gene function within their genomic context in ant lineages that do not exhibit reproductive behavior in workers. This method can be leveraged to investigate the large odorant receptor (OR) gene families present in ant clades. However, ORs tend to be expressed in a tissue-specific fashion, and the capability of dsRNA to achieve knockdown of genes exhibiting localized expression remains uncertain. In this study, we use fluorescently labelled dsRNA to track the spread of dsRNA through the worker body, qPCR to verify that dsRNA can knockdown gene expression in the antennae, and behavioral trials to verify that knockdown of ORs affects nestmate recognition. Our results show ingested dsRNA is able to reach the antennae and knock down odorant coreceptor (*orco*) expression in the antennae. We determined that orally-administered dsRNA is capable of being spread systemically and knocking down tissue-specific genes. These findings show that RNAi may be useful in investigating the influence of ORs in eusocial insect nestmate recognition.

## 1. Introduction

Olfaction is the primary method through which insects to locate food, mates, and sites to lay their eggs [1]. In order to better detect and respond to odors, insects have evolved a complex odorant reception apparatus that involves numerous proteins for odorant transport and perception [2]. Research in the *Drosophila* model system has described a system of odorant binding proteins that shuttle odorants through the sensillar lymph to odorant reception complexes in the neuron membrane that bind to these odorants and induce signal transmission to the antennal lobe [2,3]. These complexes likely consist of heterotetramers consisting of two copies of a tuning OR gene that confers sensitivity to specific odorants, and two copies of the odorant coreceptor (*orco*) [3]. While most insects carry only one copy of the *orco* gene, the amount of tuning OR genes varies wildly by insect group, going as low as three in Odonata and into the hundreds in Formicidae. This massive expansion of ORs is thought to be one of the factors facilitating the development of eusociality in ant species [4–6].

One of the primary advantages that allow eusocial species to function in diverse ecosystems is the ability to form colonies composed of many adults that work together for the colony’s benefit. Nestmate recognition through pheromone signaling is the backbone of colony formation and maintenance in eusocial insect species [7–9]. Workers rely on primarily hydrocarbon-based odorant cues to distinguish nestmates from non-nestmate workers [7]. These hydrocarbons are detected by the odorant receptors present in the membrane of antennal sensillar neurons [2]. The identity of specific ORs involved in nestmate recognition is unknown, but an expansion of nine-exon ORs in Formicidae is considered a likely group [4,10]. In particular, some of these nine-exon ORs show antennae-biased gene expression in *L. humile* [11]. Much work has been done to investigate how cuticular hydrocarbons (CHCs) and ORs influence nestmate recognition in ant species through a variety of methods, such as environmental modification and ligand-binding assays [12–14]. However, the contribution of individual tuning ORs to nestmate recognition remains unclear, due to difficulties in studying gene function in eusocial species.

Thanks to the development of mutagenesis tools such as *CRISPR-Cas9*, functional characterization of genes has become much more approachable in recent years [15]. However, these tools rely on reproduction in the mutants to generate a population that can be used for experimentation. While some ant species are capable of worker reproduction, obligate worker sterility in many ant species renders this tool impractical for functional characterization [16,17]. Insertion of genes into model organisms for study, such as the Gal4/UAS or empty neuron systems in *Drosophila*, remains an option in these cases, but the process is time-consuming and removes the gene from its organismal context [18–20]. However, RNA interference (RNAi) is a potentially viable alternative that requires much less effort to set up experiments [21]. RNAi techniques have already shown their efficacy in functional characterization of olfactory genes using both injection and ingestion routes of entry, such as how olfactory genes influence host-finding behavior in mosquitoes or alarm behavior in ants [22–25]. Prior work has identified some likely candidate ORs that may be involved in nestmate recognition [11]. In this paper, we examine the potential of dsRNA for use in investigating OR function in ants through tracking the systemic spread of oral dsRNA, verifying antennal gene knockdown, and identifying changes in nestmate recognition post-knockdown.

## 2. Methods

### 2.1. Colony collection and maintenance

Portions of two *Linepithema humile* colonies exhibiting aggression towards each other were collected from Winston-Salem and Raleigh, North Carolina via nesting substrate collection and brought to the lab. The colonies were extracted from the nesting material and allowed to move into artificial nests consisting of foil-covered 15x150mm test tubes. Once extracted, the colonies were transferred to Fluon-lined trays and kept under 80 ± 2°F, 50 ± 5% relative humidity, and 14:10 light:dark cycle. These colonies included multiple queens and brood, and were kept in the lab long-term for all assay work. Colonies were provided with water, 20% sucrose solution (w/v), and freshly killed American cockroaches. For dsRNA feeding trials, foraging workers were collected from both colonies and transferred to Fluon-lined trays and provided with foil-wrapped 10x75mm test tubes nests half-filled with water. Workers were considered foraging if they were gathered around food or water sources, or travelling from these sources to the test tube nests. These workers were starved for 24 hours before experiments were started, at which point they were fed daily with 10% sucrose solution dosed with dsRNA.

### 2.2. dsRNA synthesis and administration

Worker mRNA was extracted using an SV Total RNA Isolation Kit (Z3101, Promega, Madison, WI). cDNA was generated using a SensiFAST cDNA Synthesis Kit (BIO-65054, Meridian Bioscience, Memphis, TN). DNA was extracted from *L. humile* workers using a DNEasy Blood and Tissue Kit to generate dsRNA targeting non-coding DNA (69504,Qiagen, Germantown, MD). T7 PCR products were generated using a SensiFAST SYBR No-ROX Kit (BIO-98050, Meridian Bioscience, Memphis, TN). dsRNA was generated from T7 Products using a Durascribe T7 Transcription Kit (DS010925, Biosearch Technologies, Middleton, WI). Primer–BLAST was used for designing primers for dsRNA synthesis and verification qPCR and ensuring target specificity [26]. Separate pairs of dsRNA and verification primers were designed to ensure qPCR verification of knockdown did not erroneously amplify extant dsRNA. dsRNA yield and quality was measured using a NanoDrop 2000C spectrophotometer (ND-2000C, ThermoFisher, Waltham, MA). Gel electrophoresis was used to verify dsRNA primer sets generated correct dsRNA molecules for RNA interference. dsRNA used in histological work was treated with *Silencer* siRNA Labeling Kit with Cy3 dye (AM1632, ThermoFisher Scientific, Waltham, MA). dsRNA was prepared for trials by mixing 1:1 with autoclaved 20% sucrose solution (w/v) to create a 10% sucrose working solution. The working solution was aliquoted into PCR tubes and stored at 4^o^C until needed.

### 2.3. Histology

Foraging workers were collected from laboratory colonies and kept in Fluon-lined plastic trays. These workers were provided artificial nests consisting of 10x75mm test tubes half-filled with water and wrapped in aluminum foil to provide darkness preferred by the ants. Several dozen workers were starved for 24 hours, then provided with 100 µl doses of 10% sugar water dosed with fluorescent-tagged dsRNA at 1 mg/mL concentration every other day for 6 days. Twenty-four hours after initial dosing, 5 workers were removed from the tub every day and stored in RNALater (AM7021, ThermoFisher, Waltham, MA). After 6 days, workers were taken from the RNALater and incubated for 48 hours in 10% paraformaldehyde in aqueous solution and submitted to the Purdue Histology Core for processing and visualization. Samples were processed through an ethanol gradient (100/95/80/75%) for dehydration then cleared with xylene and infiltrated with paraffin before being embedded in a block for sectioning. Tissues were sectioned into 4μm slices using a Leica HistoCore AUTOCUT (Leica Biosystems, Deer Park, IL) onto charged slides and deparaffinized using three xylene treatments and two 100% ethanol treatments before being rehydrated using DI water. The slides were stained with Alexa Fluor 555 secondary antibody and DAPI, then dehydrated and cover slips applied. The slides were then scanned at 20x for fluorescence using a Leica Aperio imaging system. Presence of dsRNA was verified in tissues based on the presence of orange signal in the sections from the Cy3 dye.

### 2.4. dsRNA efficacy testing

**Fig 1:**
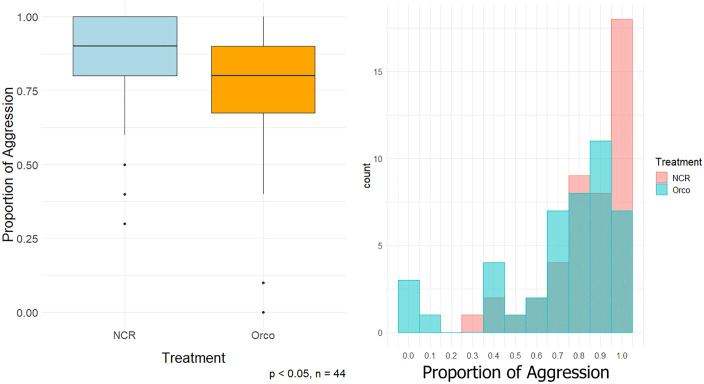
Aggression of 0.9 µg/µL *orco* dsRNA vs. NCR dsRNA. (A): Aggression assay boxplot comparing average aggression between ants treated with *orco* dsRNA vs NCR dsRNA. Aggression was calculated by scoring ten 1:1 interactions as described in section 3.4 and taking the average of the scores before analyzing resulting data using a Mann-Whitney U-test. Ants treated with *orco* showed a significant reduction in cross-colony aggression compared to NCR-treated ants (p < 0.05, n = 44). (B): Aggression assay histogram comparing the distribution of proportion of aggression responses between ants treated with *orco* dsRNA vs NCR dsRNA. Aggression patterns shifted broadly less aggressive in ants treated with *orco* dsRNA than NCR-treated ants.

**Fig 2:**
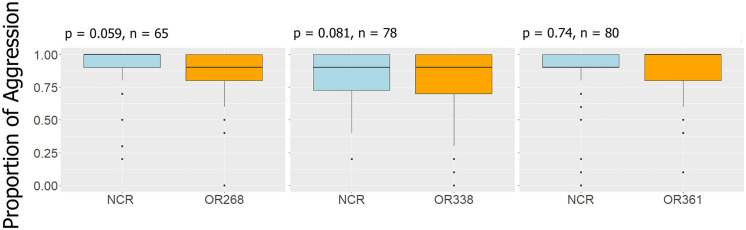
Aggression assay boxplots of 1 µg/µL OR dsRNA vs. NCR dsRNA. ORs *LhOr268* (n = 65, p = 0.059) and *LhOr338* (p = 0.08, n = 78) show potential mild loss in aggression in OR treated trials, while OR *LhOr361* (n = 80, p = 0.74) shows no difference in aggression. Aggression data was calculated and analyzed similarly to *orco* trial. Efficacy of tuning OR knockdown on aggression is uncertain and observed changes in behavior tend to be very small.

Approximately 120 foraging workers were collected from laboratory colonies and kept in Fluon-lined plastic trays. Four of these replicates were taken for each combination of origin colony (Winston-Salem or Raleigh) and dsRNA (OR or non-coding region). These workers were provided foil-wrapped 10x75mm test tubes as shelter with water sequestered behind a cotton ball and starved for 24 hours, then provided with daily aliquots of 50 µl of sugar water dosed with dsRNA that targeted either an odorant receptor gene identified in prior literature as a nine-exon OR gene expressed solely in the antennae, or a non-coding region of the *L. humile* genome (See S4 Table for concentration and target accession number) for 6 days (11). Target dsRNA concentration of 1 μg/μL was chosen based on prior literature conducting RNAi using ingested dsRNA in ants [27]. Target genes were chosen based on prior studies showing nine-exon clade status and antennae-specific gene expression [10,11]. After 6 days, treated workers were subjected to 1:1 aggression assays using a 1–4 aggression scale (1- Touch, 2 – Flee, 3 – Biting/Lunging, 4 – Wrestling) [28]. One worker from each colony colony (Winston-Salem and Raleigh) were removed from their trays via paintbrush and transferred to a Fluon-lined 35x10mm Petri dish where they were allowed to interact while being monitored by a single researcher. The first ten interactions between these workers were scored using the aggression scale in each worker pairing, at which point each worker was transferred to a separate Fluon-lined tray to ensure they were not reused. The amount of 1:1 trials conducted for each OR trial (n = 44–78) varied based on the amount of surviving workers after the six-day dosing period (Figs 1-2). Aggression levels 1 and 2 were considered Not Aggression (0) and levels 3 and 4 were considered Aggression (1). The resulting binary data was then used to generate a proportion of aggressive behavior on a 0–1 scale using the ten scored interactions from each 1:1 pairOnce aggression trials were completed, the workers were immersed in RNALater at 4^o^C for later dissection.

Both antennae were removed from workers that completed aggression trials and stored in RNALater at 4^o^C during sample collection. For each sample, forty antennae were collected from twenty workers and stored as a single pooled sample in RNALater at 4^o^C. Once sample collection was complete, RNALater was pipetted off and RNA was extracted using an SV Total RNA Isolation Kit, and cDNA was generated using a SensiFAST cDNA Synthesis Kit, with input RNA standardized based on input tissue. qPCR was conducted using a SensiFAST SYBR no-ROX Kit on a CFX96 Real Time System/C1000 Thermal Cycler (Bio-Rad Laboratories, Hercules, CA). qPCR protocol was based on the 3-step cycling recommended by SYBR kit, with a 65^o^ melt temperature and 55-95^o^ melt curve. Two technical replicates were done for each sample, and β-actin was used as the control gene based on prior work [29–32]. Gene expression was calculated using the ΔΔCt method after confirming primer efficiency was within acceptable levels [33]. Statistical analyses of aggression assay and qPCR Ct data were conducted via Mann-Whitney U test using *R* (4.1.0) and *RStudio* (2023.03.1 Build 446).

## 3. Results

### 3.1. Fluorescence localization

**Fig 3:**
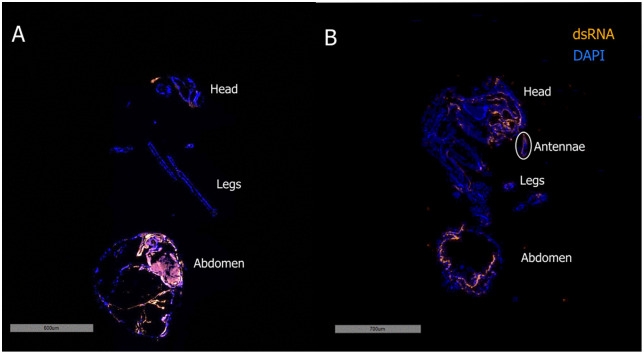
Confocal microscopy of *L. humile* longitudinal sections. (A): Day 5 longitudinal image with fluorescence showing dsRNA (Cy3-tag/orange) and cell nuclei (DAPI/blue). Very strong orange signal in abdominal tissues indicate dense concentrations of dsRNA present in worker midgut. (B): Day 1 longitudinal image with fluorescence showing dsRNA (Cy3-tag/orange) and cell nuclei (DAPI/blue). dsRNA signal is found in all major body regions of worker, indicating that dsRNA is capable of crossing midgut into hemolymph and spreading throughout body within twenty-four hours of ingestion.

The Cy3 signal (orange) bound to the dsRNA was strongest in the abdominal tissues, indicating that dsRNA has difficulty exiting the midgut into the hemolymph (Fig 3A). However, dsRNA can cross the gut lumen, enter the hemolymph, and reach all major body regions of worker ants within twenty-four hours of ingestion, based on the presence of Cy3 signal in the antennae, head, leg, and thoracic tissues (Fig 3B).

### 3.2. Gene expression knockdown

**Fig 4:**
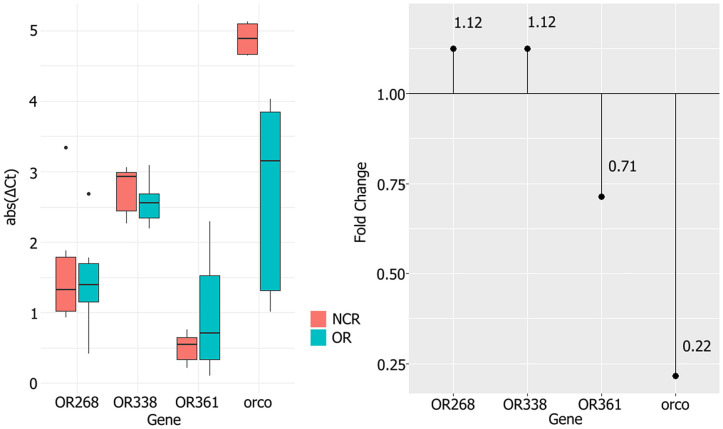
(A) Boxplots comparing ΔCt values of OR genes between control (non-coding region) and experimental ingested RNAi treatments. Statistical analysis of OR data was conducted via Mann-Whitney U Test on ΔΔCt data. The *orco* gene shows a change in ΔCt values after treatment with dsRNA targetting the *orco* gene (W = 0, p < 0.05, n = 5). Treatments targetting three tuning OR genes (*LhOr268, LhOr338, LhOr361*) show no significant change in ΔCt values compared to NCR controls (W = 19,18,25; p > 0.05, n = 6, 5, 8). (B). Plot showing fold-change values for the four tested OR genes in response to exposure to dsRNA targeting the OR gene. The *orco* gene shows a large reduction in expression in *orco* after exposure to dsRNA targetting *orco* gene. The three tuning OR genes, show little fold change.

The ingestion of *orco* dsRNA resulted in significant reductions in the expression of *orco* gene in the antennae compared to noncoding dsRNA (NCR) (W = 0, p < 0.05, n = 5) (Fig 4). However, none of the dsRNA treatments targeting tuning ORs produced a significant reduction in gene expression (W = 19, 18, 25; p > 0.05, n = 6, 5, 8) (Fig 4).

### 3.3. Behavioral assays

**Fig 5:**
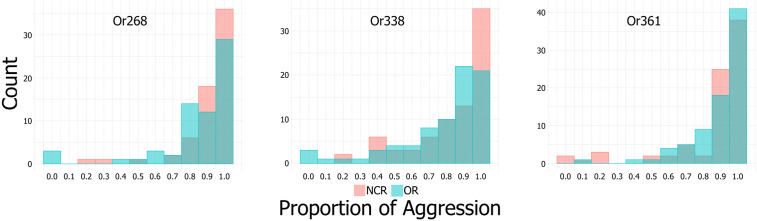
Aggression assay histograms of 1 µg/µL OR dsRNA vs. NCR dsRNA. ORs *LhOr268* and *LhOr338* show similar pattern shift in aggression reduction as displayed in *orco*-treated ants. OR *LhOr361* showed no meaningful shift in patterns of aggression between ants treated with *LhOr*361 dsRNA and NCR dsRNA.

The ingestion of *orco* dsRNA also significantly reduced intercolony worker aggression (0.8 on a 0−1 aggression scale) when compared to workers fed non-coding region dsRNA (0.9 on a 0−1 aggression scale) (Fig 1A). This reduction in aggression is driven by a pattern of slight loss in aggression (0.1–0.3 reduction) in many treated ants versus control (Fig 1B). Aggression assays conducted on ants treated with dsRNA targeting *LhOr268* (0.85 vs 0.91, p = .059) and *LhOr338* (0.78 vs 0.83, p = .081) show a small but insignificant loss in aggression while *LhOr361* showed no change in aggression (.089 vs.086, p = 0.74), though none of the tuning OR trials were significant (Fig 2). Similar to the *orco* aggression trial, The reduction in aggression in *LhOr268* and *LhOr338* treatments versus their NCR controls is driven by a pattern of small reduction in aggressive behavior (0.1–0.2 reduction) across numerous 1:1 worker pairings (Fig 5).

## 4. Discussion

Results demonstrate that dsRNA can cross the midgut tissue and inhibit gene expression even in genes that only show expression in tissues at the body periphery, as exhibited by dsRNA yielding a reduction in *orco* gene expression in antennal tissues. This reduction,in *orco* expression produced a reduction in aggressive behavior similar in effect to the total loss of aggression when treated with chemical-based *orco* antagonists, although the magnitude of effect from dsRNA feeding was much smaller [14]. The small effect size on aggression resulting from dsRNA feeding matches other dsRNA feeding work on *L. humile* immune response, where targeting immune genes resulted in small changes in pathogen load [34]. Targeting tuning ORs might produce a mild decrease in aggression in some tuning ORs, but evidence remains uncertain. Given the size of the nine-exon OR clades thought to be involved in nestmate recognition, a mild reduction of aggression in response to loss in normal function of a single tuning OR would be expected, potentially generating an effect too small to measure with aggression assays. However, qPCR showed no detectable knockdown of expression of these genes. There are several potential explanations for this lack of knockdown. First, our *orco* dsRNA molecule was longer (461 bp) than the dsRNA molecules targeting the tuning ORs (150–280 bp). Prior work in other insect systems indicates that dsRNA effectiveness increases with molecule length [35–37]. Treatments with longer dsRNA molecules may prove more effective at inhibiting tuning receptor function. Second,

it is possible that the knockdown effect on *orco* was detectable due to the higher overall expression, while the low levels of tuning OR expression under control conditions may have made it too difficult to detect dsRNA-induced knockdown. Third, evidence thus far indicates that tuning ORs are expressed within a small number of sensilla [38]. Given that much of the dsRNA seems to stay in the midgut, there might be insufficient transfer into the hemolymph to saturate all antennal tissues and ensure the correct ORNs uptake the dsRNA, in which case more research is needed into factors that influence transfer efficiency of dsRNA out of the midgut and into target tissues, such as the presence of dsRNA-targeting nucleases that impair RNAi in other insect groups, identifying the genes associated with dsRNA uptake (such as *Sid* and *Chc* genes implicated in other insects), and encapsulation techniques to protect dsRNA integrity during transit to the target site [39–44]. Alternatively, extending the duration of feeding experiments as has been done in prior work could allow for more time for dsRNA to inhibit OR expression [34]. Finally, there is a possibility that the knockdown effect of the dsRNA is being masked. Gene expression work in the clonal raider ant, *Ooceraea biroi*, determined that ORs in tandem arrays are transcribed, but only the first gene is exported to the cytoplasm for expression [45]. If OR expression in *L. humile* works similarly, the abundance of OR transcripts in the ORN nucleus may be out of reach of the RISC complex and therefore masking the effects of gene knockdown when conducting qPCR analysis. Future work could avoid this potential issue by investigating a more quantitative way to measure the effect of tuning OR knockdown would be to conduct coupled gas chromatography-electroantennography (GC-EAG) on treated antennae compared to control, to look for changes in antennal detection of cuticular hydrocarbon components [46,47]. Similar work successfully verified knockdown of odorant binding proteins in *S. invicta* using injected dsRNA via EAG [24]. This analysis would also provide a more practical method for characterizing the CHC ligands that bind to a specific odorant receptor. Current methods for investigating OR function generally rely on mutagenesis, either using CRISPR to generate a knockout mutant, or through introducing the gene into *Drosophila* fly lines, *Xenopus* oocytes, or human embryonic kidney (HEK293) cell lines [17,18,48,49]. RNA interference represents a much less labor- and time-intensive process for conducting functional characterization of genes and allows for the investigation of gene function in its original context. Prior work investigating other odor-sensing genes in insects has shown that both injected and fed dsRNA can be used to investigate antennal gene function, and orally administered RNAi has already shown effectiveness in *L. humile* [23–25,34] This is particularly necessary in a eusocial insect like the Argentine ant, where the reproductive division of labor makes cultivating a viable population of mutants incredibly difficult. Additionally, since learning colony odor takes place upon pupal eclosion, any attempt to identify the influence of an OR on nestmate recognition via mutagenesis is not possible [7]. Therefore, RNAi-mediated knockdown of OR expression is currently the only potential method that can allow us to investigate how individual ORs regulate nestmate recognition, and our work shows that ingested dsRNA is capable of knocking down OR expression in adults. While we weren’t able to verify confirm knockdown of tuning OR expression, our initial results confirming that dsRNA is capable of affecting OR expression are encouraging. With further optimization of RNAi methodologies in ants, dsRNA could prove to be a valuable tool when investigating how OR function influences colony-level behavior such as foraging, nursing, and nestmate recognition in ants. In a more practical area, dsRNA has been suggested as the next frontier of pest management [41,50–52]. Work is already underway investigating immune genes as potential targets for *L. humile* population control and given the important that ORs have in nestmate recognition and detection of reproductive cues, we could develop dsRNA-based control agents that could disrupt invasive *L. humile* populations, as well as other invasive ant species, without damaging nontarget species [34].

## Supporting information

S1 Table Table containing the Aggression data used to determine if RNAi showed behavioral effects. (XLSX)

S2 Table Table containing primers for dsRNA generation and qPCR verification of RNAi targets. (XLSX)

S3 Table Table containing Ct, ΔCt, ΔΔCt, and fold-change (FC) data used for determining whether RNAi resulted in change in gene expression for target genes. (XLSX)

S4 Table Results of RNAi for each target, including ΔCt, ΔΔCt, and fold-change (FC) data. ΔCt value indicates the deviation of mean Ct value of test gene (OR gene of interest) from the control gene (β-actin). ΔΔCT value indicates deviation in mean ΔCt value of experimental treatment (OR dsRNA) from control treatment (NCR). Only *orco* was found to exhibit reduction in gene expression in response to dsRNA ingestion (Mann-Whitney U-Test, p < 0.05).
(XLSX)
